# Histone H3K56 Acetylation, Rad52, and Non-DNA Repair Factors Control Double-Strand Break Repair Choice with the Sister Chromatid

**DOI:** 10.1371/journal.pgen.1003237

**Published:** 2013-01-24

**Authors:** Sandra Muñoz-Galván, Sonia Jimeno, Rodney Rothstein, Andrés Aguilera

**Affiliations:** 1 Centro Andaluz de Biología Molecular y Medicina Regenerativa (CABIMER), Universidad de Sevilla–CSIC, Sevilla, Spain; 2 Department of Genetics and Development, Columbia University Medical Center, New York, New York, United States of America; NYU Medical Center, United States of America

## Abstract

DNA double-strand breaks (DSBs) are harmful lesions that arise mainly during replication. The choice of the sister chromatid as the preferential repair template is critical for genome integrity, but the mechanisms that guarantee this choice are unknown. Here we identify new genes with a specific role in assuring the sister chromatid as the preferred repair template. Physical analyses of sister chromatid recombination (SCR) in 28 selected mutants that increase Rad52 foci and inter-homolog recombination uncovered 8 new genes required for SCR. These include the SUMO/Ub-SUMO protease Wss1, the stress-response proteins Bud27 and Pdr10, the ADA histone acetyl-transferase complex proteins Ahc1 and Ada2, as well as the Hst3 and Hst4 histone deacetylase and the Rtt109 histone acetyl-transferase genes, whose target is histone H3 Lysine 56 (H3K56). Importantly, we use mutations in H3K56 residue to A, R, and Q to reveal that H3K56 acetylation/deacetylation is critical to promote SCR as the major repair mechanism for replication-born DSBs. The same phenotype is observed for a particular class of *rad52* alleles, represented by *rad52-C180A*, with a DSB repair defect but a spontaneous hyper-recombination phenotype. We propose that specific Rad52 residues, as well as the histone H3 acetylation/deacetylation state of chromatin and other specific factors, play an important role in identifying the sister as the choice template for the repair of replication-born DSBs. Our work demonstrates the existence of specific functions to guarantee SCR as the main repair event for replication-born DSBs that can occur by two pathways, one Rad51-dependent and the other Pol32-dependent. A dysfunction can lead to genome instability as manifested by high levels of homolog recombination and DSB accumulation.

## Author Summary

Double-strand breaks (DSBs) are among the most dangerous DNA lesions and can lead to genomic instability, a process associated with cancer and hereditary diseases. An important source of DSBs is replication, Sister Chromatid Recombination (SCR) being the main mechanism for DSB repair in dividing eukaryotic cells. SCR repair is error-free and uses the sister chromatid as template, generating an identical DNA sequence and therefore preventing genomic instability. In this work, we use an inverted-repeat assay with which we can physically detect SCR intermediates generated by the repair of a replication-born DSB. We hypothesized that SCR defects can result in an increase of recombination with the homologous chromosome, so we assayed SCR in 28 mutants previously described to increase homolog recombination. Our results describe 8 new genes involved in SCR, including functions such as histone acetylation/deacetylation, SUMO-Ubiquitin metabolism, and stress response, as well as an allele of *RAD52*. This demonstrates the importance of the choice of the sister chromatid as template for DSB repair and provides a broad vision of SCR as a tightly regulated process essential for genome integrity.

## Introduction

In eukaryotic cells, DSBs can be repaired either by homologous recombination (HR) or by non-homologous end joining (NHEJ). From these, only HR with the sister chromatid ensures maintenance of genome integrity, sister chromatid recombination (SCR) being the preferred mechanism of DSB repair in mitotic cells [1]–[3]. As any other HR event, SCR requires the action of DSB repair genes, many of them constituting the *RAD52* epistasis group [4]. Given the relevance of SCR in the repair of replication-born DSBs as well as that the sisters are the products of replication, it is expected that a number of specific functions should contribute to SCR with little impact in the repair of DSBs by HR with ectopic DNA sequences or homologous chromosomes. Thus, in addition to DSB repair genes, other functions contribute to hold the sister chromatids together and to facilitate SCR *versus* HR with the homologous chromosome, such as cohesins or the Smc5-Smc6 complex [3], [4]. However, we still have very little knowledge of SCR specific functions.

One key aspect of DNA replication is chromatin duplication, which implies the assembly of nascent nucleosomes on the sister chromatids as they are generated (reviewed in [5]). However, packaging of DNA into chromatin may result in a barrier to all DNA transactions. As a result, eukaryotic cells have specialized machinery to modify the histones to facilitate DNA metabolic functions. One such type of modification is acetylation of lysines, which play different roles in transcription, DNA repair and replication. An acetylatable residue particularly important in cell cycle progression is Histone H3 Lysine 56 (H3K56). In budding yeast, the transient acetylation of H3K56 (H3K56ac) occurs on newly synthesized H3 molecules by Rtt109 acetyl-transferase, and facilitates their deposition onto newly replicated DNA during S phase. However, this acetylation disappears rapidly by the action of sirtuins Hst3 and Hst4 when cells enter G2/M [6], [7].

Completion of H3K56ac-dependent chromatin reassembly is likely required for resumption of cell proliferation after DNA repair [8]. H3K56ac is conserved in human cells, where it also appears to be regulated in a DNA damage-dependent manner [9]–[11]. Interestingly, yeast strains lacking an acetylatable lysine 56 show genetic instability and sensitivity to a subset of genotoxic agents, such a camptothecin (CPT) [6], [12], a phenotype possibly due to a key role of this modification in nucleosome assembly following DNA replication and DNA repair [8].

Even though a large body of data shows that defective replication underlies the high levels of DNA instability associated with chromatin remodeling mutants, their pattern of genome instability suggests that additional mechanisms yet unexplored may play a role in this process. This view is more evident in light of a genome-wide screen in *S. cerevisiae* for mutants exhibiting high levels of Rad52 foci, a mark of DSB-repair centers. Such a screen identified *hst3Δ*, which causes a DSB repair defect and increased rates of HR between homologous chromosomes but normal levels of direct-repeat recombination [13]. This phenotype may be due to replication failures, but it is also compatible with a defect in SCR. Interestingly, this screen also identified a number of mutants with similar phenotypes that are potential candidates to be impaired in SCR that have not been further analyzed. Notably, the role of Rad52 in DSB repair and spontaneous HR can also be separated, as shown by the old *rad52-2* mutation [14] or those grouped as the class C mutants of *RAD52*, which are sensitive to γ-radiation but maintain wild-type levels of mitotic HR [15].

Using a physical assay for the kinetic analysis of the repair of replication-born DSBs generated at a 24-bp mini-HO site [3] we have identified new factors that promote SCR among 27 mutants previously identified as inter-homolog hyper-recombinant and accumulating Rad52 foci [13], including Wss1, a SUMO or Ub-SUMO protease [16], and several proteins involved in chromatin remodeling, such as Ahc1 (structural subunit of ADA histone acetyltransferase complex) [17] and Hst3 or Rtt109, involved in acetylation/deacetylation of H3K56. In addition we show that the class C mutant *rad52-C180A* is also impaired in SCR. Taken together, our results suggest that a broad range of factors regulate the choice of the sister chromatid as the template for the repair of replication-born DSBs at an early step of the HR reaction, guaranteeing genome integrity.

## Results

### New functions specifically required for the choice of SCR as the repair mechanism for replication-born DSBs

**Figure 1:**
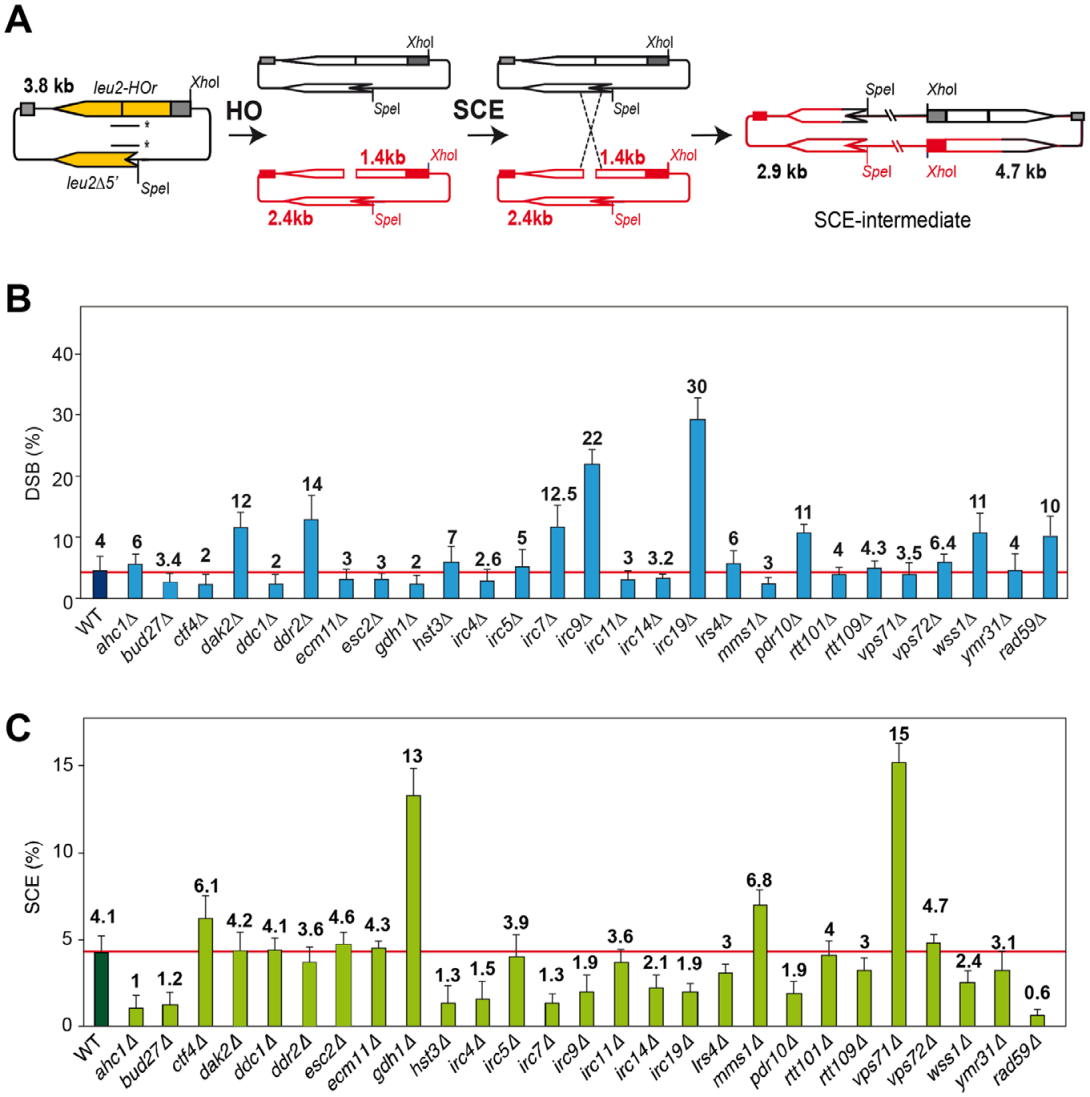
Molecular analysis of SCR in 27 hyper-recombination mutants. (A) Scheme of plasmid pRS316TINV used for the physical monitoring of SCR of a replication-born DSB. Fragments generated after *Xho*I-*Spe*I digestion, detected with a *LEU2* probe (line with asterisks) are indicated with their corresponding sizes. SCR is monitored by appearance of the 4.7-kb fragment, the only one unequivocally occurring via SCR. (B) Quantification of DSB 2.4- and 1.4-kb fragments after 6 hours of HO activation in galactose. (C) Quantification of the SCR 4.7-kb fragment after 6 hours of HO activation in galactose. All strains, isogenic to W303, were transformed with pRS413GALHO harboring the HO endonuclease gene under the control of the *GAL10* promoter, and pRS316TINV. Average and standard deviations of three samples from each genotype are plotted. Additional results are shown in Figures S1, S2, S3.

A physical assay to monitor the repair by SCR of a single DSB generated during replication [3], [4] has been used to determine the efficiency of SCR in 27 mutants previously identified by their high levels of Rad52 foci increase and inter-homolog recombination. These mutants define genes affected in diverse cellular mechanisms, such as replication, DNA repair, DNA damage response, chromatin remodeling and genes with unknown functions termed *IRC* (*i*ncreased *r*ecombination *c*enters) [13], Figure 1). In addition, we included the *rad52-C180A* mutant sharing the phenotype of increased levels of inter-homolog recombination and DSB repair defect [15]. The SCR assay is based on a circular minichromosome, pRS316-TINV, harboring an internal mini-HO site, which is cleaved mainly in one strand producing 10% DSBs during replication. Upon HO induction, a DSB occurs mainly in only one chromatid, the other remaining intact and available for repair (see Figure 1A). Although this assay has been used mostly to monitor unequal SCR events, it has been demonstrated that it is an accurate indicator of the proficiency in total SCR [3], [4], [18]

DSB and SCR intermediates were assayed in isogenic W303 strains carrying an endogenous *LEU2* sequence (*leu2-3,112* allele) and expressing the HO endonuclease from a plasmid. Kinetics analyses during 9 h after induction of HO revealed that 14 mutants were impaired in SCR (Figure 1C, Figures S1 and S3), including the previously reported DSB repair mutant *rad59* [18]. The rate of DSB accumulation was differentially affected, showing an increased accumulation in *dak2*, *ddr2*, *irc7*, *irc9*, *irc19*, *pdr10* and *wss1*, consistent with a severe defect in SCR repair in five of them (*irc7*, *irc9*, *irc19*, *pdr10* and *wss1*), while in *dak2* and *ddr2* repair was proficient (Figure 1B and Figure S2).

**Figure 2:**
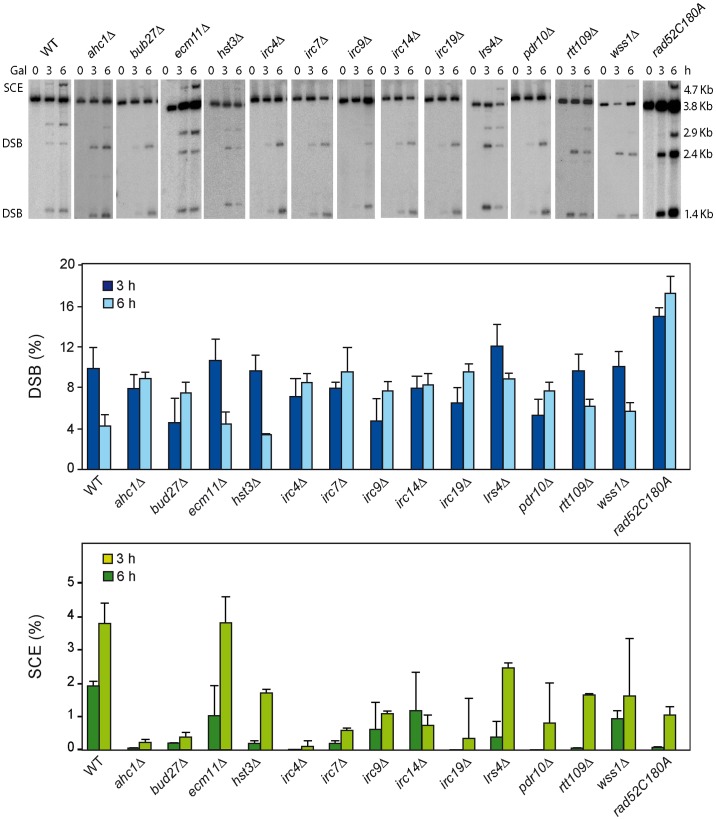
Molecular analysis of SCR in 13 SCR–defective mutants. Kinetic analysis of SCR in 14 mutants (WS strains isogenic to W303) pre-selected as SCR-defective candidates. Representative genomic blots and quantification of HO-induced DSBs (upper panel) and SCE recombination (lower panel) are shown. Average and standard deviations of three samples from each genotype are plotted.

As endogenous *LEU2* sequences could interfere with the SCR events, physical analysis of the newly identified mutants were performed in W303 isogenic background expressing HO from a chromosome and lacking endogenous *LEU2* sequences (WS strains). This analysis revealed that 12 mutants (*ahc1*, *bud27*, *hst3*, *irc4*, *irc7*, *irc9*, *irc14*, *irc19*, *lrs4*, *pdr10*, *rtt109* and *wss1*) were consistently impaired in SCR (Figure 2, lower panel). Notably, the *rad52-C180A* mutant was also defective in SCR (Figure 2).

**Figure 3:**
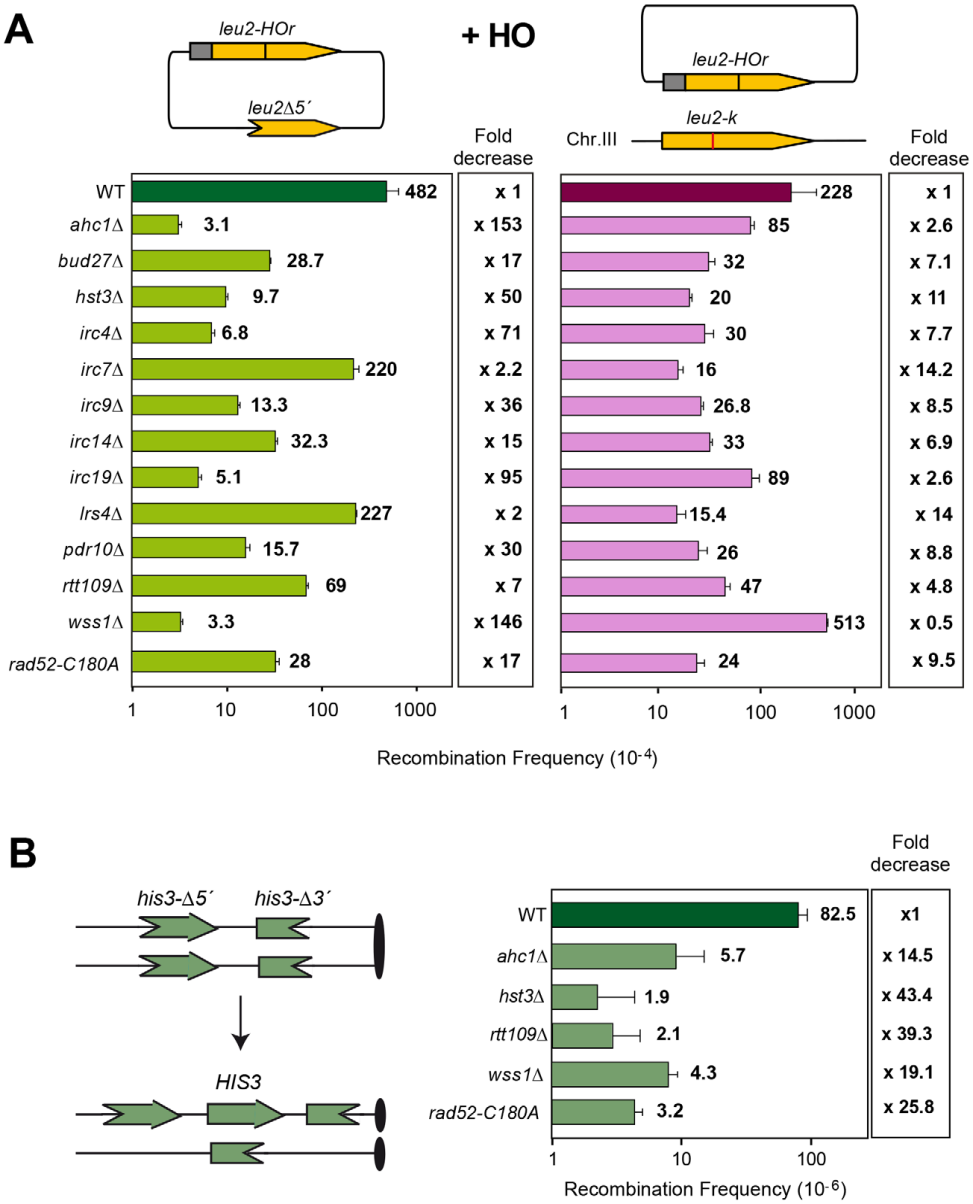
Genetic analysis of recombination in the 13 SCR–defective mutants. (A) Analysis of Leu+ intrachromosomal recombination, as an indirect measure of unequal SCR, and plasmid-chromosome recombination events after 5 hr of HO activation in 2% galactose. Values plotted for each genotype are the average and standard deviations of the median of three independent fluctuation tests (each based on 6 samples) performed with three different transformants. (B) Analysis of spontaneous SCR in the chromosomal direct-repeat system *his3-Δ5′::his3-Δ3′* in WT, *hst3Δ*, *ahc1Δ*, *rtt109Δ*, *wss1Δ* and *rad52-C180A* strains. A picture of the system and the expected His3+ recombination products are shown.

We next used a genetic analysis to compare the ability of each mutant to repair the same HO-induced replication-born DSB with either the sister or with a homologous sequence on chromosome III. As seen in Figure 3A, intrachromosomal recombination in the TINV system, which measures repair of the HO-induced replication-born DSB by several mechanisms (equal and unequal SCR and a weak contribution of intrachromatid recombination; see [3], [4]), was clearly diminished in 11 mutants (*ahc1*, *bud27*, *hst3*, *irc4*, *irc9*, *irc14*, *irc19*, *pdr10*, *rtt109*, *wss1* and *rad52-C180A*), consistent with the physical analysis. In these mutants, repair of the HO-induced replication-born DSB by plasmid chromosome recombination was also diminished, albeit to a lesser extent. We cannot discard that the topological constraints of recombination events might be different when using plasmid versus chromosome heteroalleles as template; however, so far there is no experimental evidence for such a difference. The most dramatic differences between the two systems were found in the *ahc1*, *irc19* and *wss1* mutants, which show a 95–153-fold decrease of intrachromosomal recombination but only a 0.5–3-fold decrease in plasmid-chromosome recombination (Figure 3A). The *rad52-C180A* mutant also shows a difference between both systems but less marked, with a 17-fold decrease of intrachromosomal recombination versus only a 9.5-fold decrease in plasmid-chromosome recombination. The finding that specific residues of Rad52 cause a DSB repair defect without decreasing spontaneous HR (C180 mutated to A) [15] suggests that class C mutants of Rad52 preferentially affect SCE (Figure 1, Figure 2, Figure 3, and Figure S4). Importantly our study also describes a number of new genes with specific roles in SCR, which include factors of unknown function such as Irc4, Irc9 and Irc19, the SUMO/Ub-SUMO protease Wss1 [16], the Bud27 and Pdr10 proteins involved in stress response [19], [20], as well as proteins involved in chromatin remodeling, such as Ahc1 (structural subunit of the ADA histone acetyltransferase complex), Ada2 (Transcription coactivator, component of ADA and SAGA complexes [17], [21] and Rtt109 and Hst3, involved respectively in acetylation and deacetylation of histone H3 lysine 56 (H3K56), a core domain residue that localizes at both the entry and exit points of a nucleosome [6], [12], [22]

The *hst3Δ* mutation is strongly affected in intrachromosomal SCR repeat recombination (50-fold decrease) and weakly in plasmid-chromosome recombination (11-fold decrease) while the *rtt109Δ* effect is not as pronounced in both systems. On the other hand, *wss1Δ* shows a strong and specific defect in SCR (146-fold decrease) that contrasts with an enhancement of recombination in the plasmid-chromosome system (2-fold). Further confirmation of this specific SCR defect was obtained with the previously reported *his3Δ5′-his3Δ3′* chromosomal repeat system for the genetic analysis of unequal SCR [23] in which spontaneous SCR was strongly reduced (15–39 fold) with respect to wild type in *hst3Δ* and *rtt109Δ* mutants (Figure 3B). The *ahc1Δ* mutation, affected in acetylation of different residues in transcription, has a diminished effect (14-fold), as it is also the case of *wss1Δ* (19-fold). The observation that these two mutants show a less severe phenotype in this system compared to the TINV system could be explained by the lower frequency of spontaneous events versus HO-induced ones, by the possibility that a fraction of spontaneous SCR events might not be initiated by DSBs but by ssDNA gaps or by the fact that genetically detected recombinants can also result from non-SCR events in the plasmid system. Interestingly, and consistent with a specific role in SCR observed at the physical level (Figure 2), the *rad52-C180A* mutant showed a strong decrease in spontaneous unequal SCR in the *his3Δ5′-his3Δ3′* chromosomal repeat system (26-fold), while it is proficient in spontaneous recombination between homologs [15].

### Histone H3K56 acetylation controls the choice of DSB repair template

**Figure 4:**
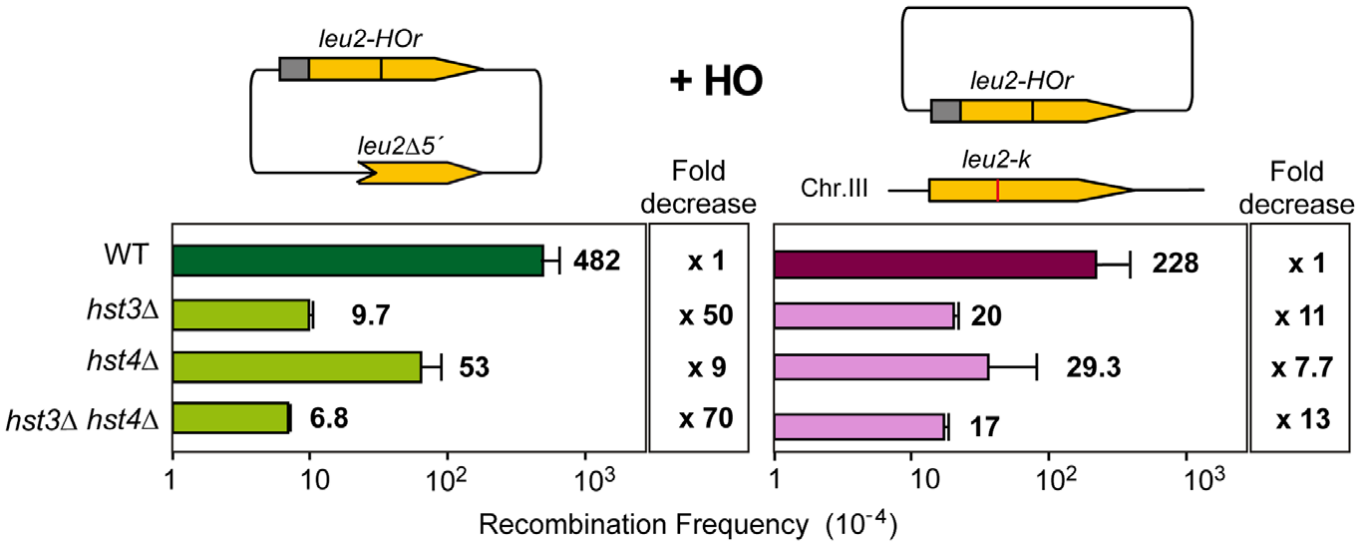
Effect of H3K56 acetylation/deacetylation on genetic SCR. Genetic analysis of unequal SCR and plasmid-chromosome Leu+ recombination events after 5 hr of HO activation in isogenic wild-type (WS), *hst3Δ*, *hst4Δ* and *hst3Δ hst4*Δ strains.

**Figure 5:**
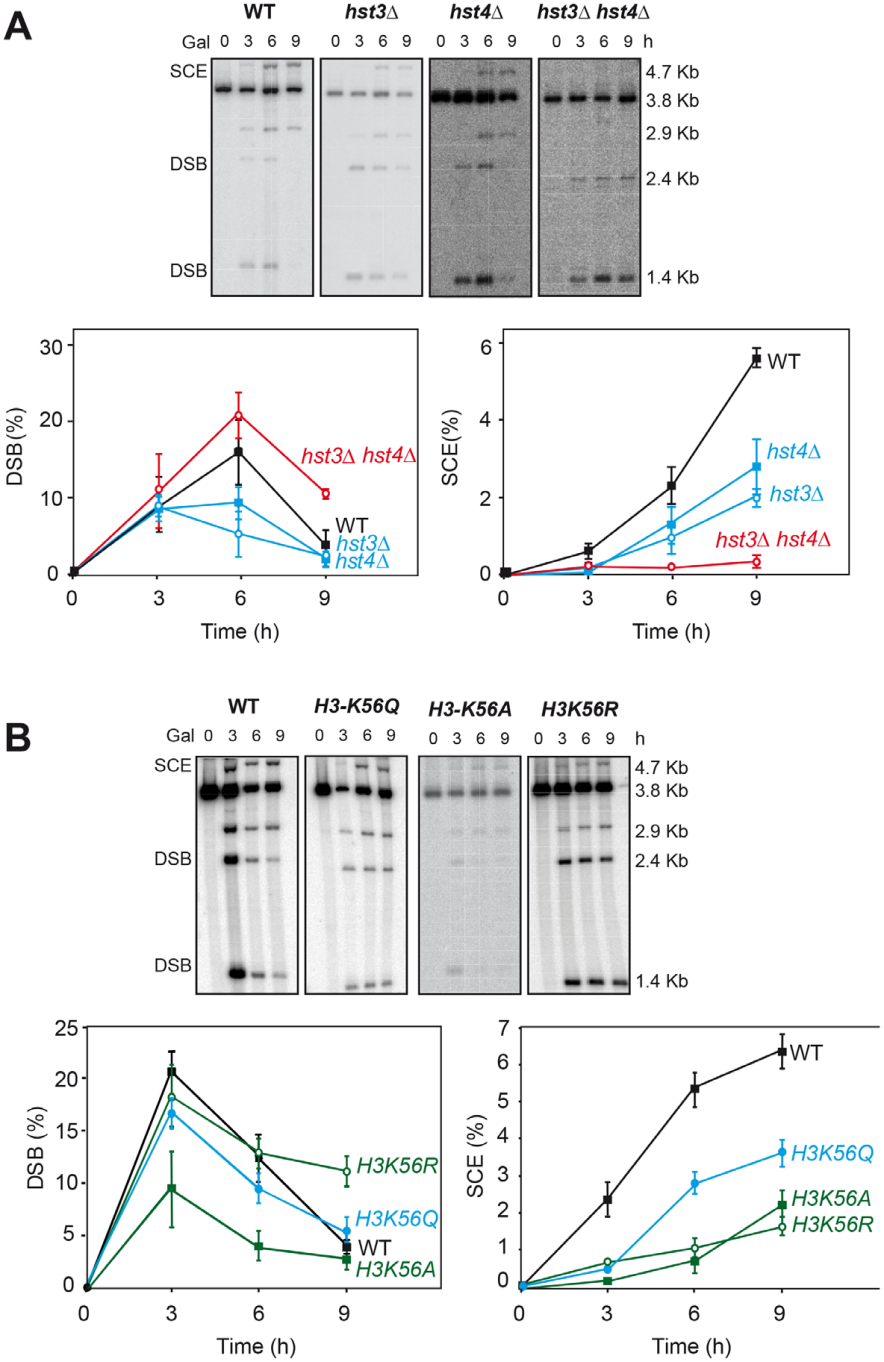
Effect of H3K56 acetylation/deacetylation on molecular SCR. (A) Physical analysis of SCR in isogenic wild-type (WS), *hst3Δ*, *hst4Δ* and *hst3Δ hst4*Δ strains after different times of HO induction (B) Physical analysis of SCR in isogenic wild-type, *H3K56R* and *H3K56Q* strains. Other details as in Figure 1.

Having identified several genes affecting chromatin remodeling, and in particular histone H3 acetylation, as important in SCR we decided to further explore the role of Histone H3 acetylation in SCR. As Hst3 is redundant with Hst4, we asked whether *hst4Δ* by itself and in combination with *hst3Δ* had a similar effect in SCR. Genetic analysis revealed that whereas *hst4Δ* decreased SCR intrachromosomal recombination 9-fold and the double *hst3Δ hst4Δ* 70-fold (Figure 4), plasmid-chromosome recombination was less affected by both mutations and to similar levels in single and double mutant combinations (8–13 fold). Physical analyses revealed that both *hst3Δ* and *hst4Δ* decreased SCR, confirming that both Hst3 and Hst4 are required for SCR, their function being redundant as deduced from the higher SCR defect of the double *hst3Δ hst4Δ* mutants (Figure 5A).

Next we asked whether the H3K56 acetylation state of chromatin plays a key role in SCR. Physical analysis of mutants *H3K56A*, *H3K56R* and *H3K56Q*, in which Lys56 was mutated to Ala and Asp, which mimic non-acetylated histone, and to Gln, which mimics hyper-acetylated histone [6], respectively, revealed that the three mutations impair SCR, the impact of *H3K56Q* being the weakest (Figure 5B). These results demonstrate that the acetylation state of H3K56 controls DSB repair by SCR, in agreement with the phenotypes of *rtt109Δ*, *hst3Δ and hst4Δ* mutants (Figure 3, Figure 4, Figure 5). Consistently, these mutants, and in particular *H3K56R*, are sensitive to replicative stress and DNA breakage inducing agents such as HU, MMS (Figure S5; [24] or camptothecin [6].

### Alternative Rad51 and Pol32-dependent SCR mechanisms in the absence of H3K56 deacetylation

**Figure 6:**
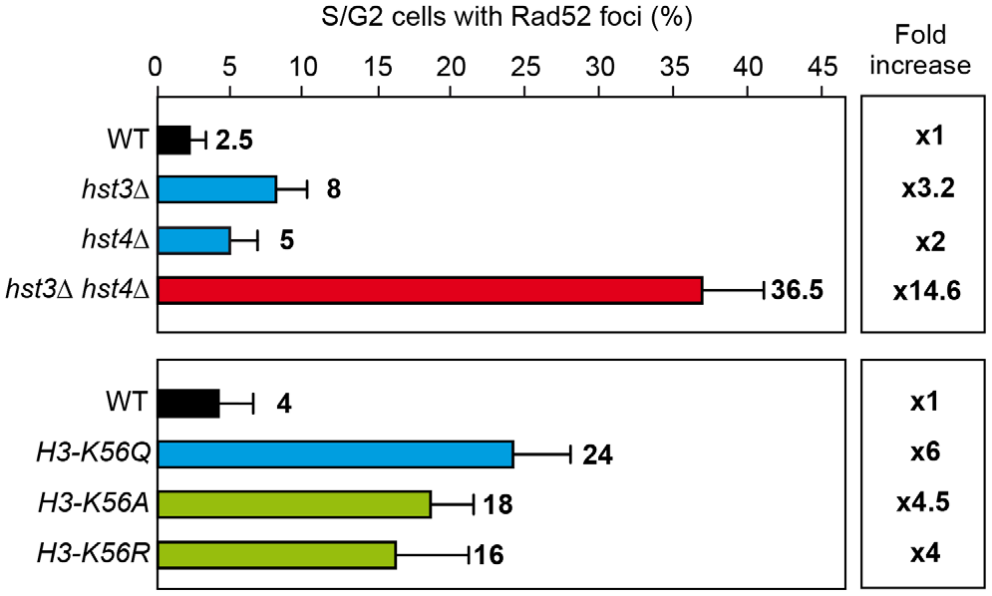
H3K56 acetylation increases DNA damage. Rad52 foci in different mutants affected in the histone H3K56 acetylation/deacetylation pattern. Average and standard deviation of two independent experiments are shown.

**Figure 7:**
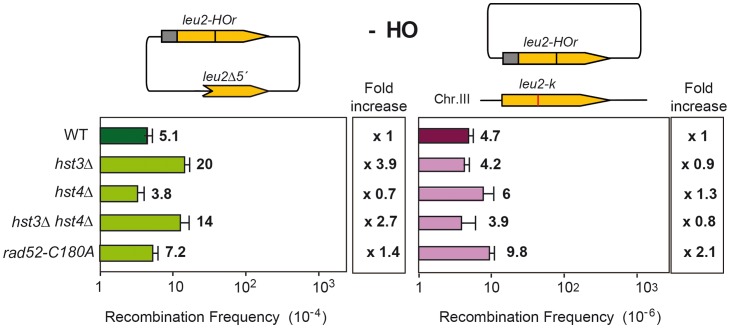
Effect of changes in the state of H3K56 acetylation on spontaneous recombination. Analysis of spontaneous intrachromosomal and plasmid-chromosome recombination in isogenic wild-type (WS), *hst3Δ*, *hst4Δ*, *hst4Δ hst3Δ* and *rad52-C180A* strains.

Spontaneous Rad52 foci accumulation and recombination were enhanced in *hst3Δ*, *hst4Δ* and synergistically in *hst3Δ hst4Δ* mutants as well as *H3K56A*, *H3K56Q* and *H3K56R* mutants confirming that all mutations cause genome instability (Figure 6). The weaker effect in *hst3Δ* and *hst4Δ* single mutants corroborates a redundant role of these two sirtuins. Spontaneous recombination was also increased in *hst3Δ* cells and not in *hst4Δ* (Figure 7), implying some functional differences between the two sirtuins consistent with other reported phenotypes [7]. Genome instability phenotypes are possibly due to the role of H3K56 modification in nucleosome assembly following DNA replication and DNA repair. Similarly, the *rad52-C180A* mutant shows no effect or a slight spontaneous hyperrecombination phenotype, consistent with previously published data for spontaneous recombination between homologs [15].

**Figure 8:**
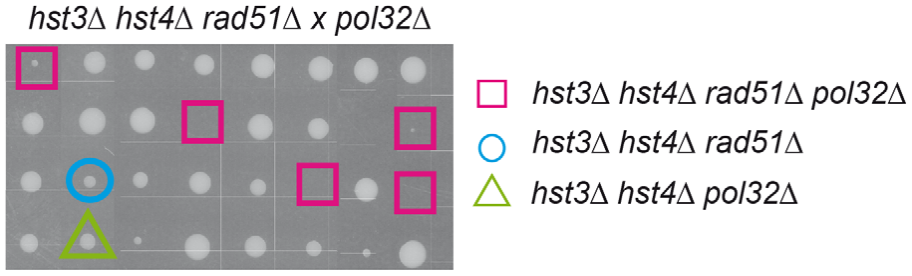
Inviability/synthetic growth defect of histone H3K56 deacetylation mutants in the absence of Rad51 and Pol32. Tetrad analysis of a *rad51*Δ *hst4Δ hst3Δ* x *pol32*Δ cross. Squares indicate quadruple mutants, which fail to grow.

Interestingly, *hst3Δ hst4Δ* double mutants are synthetic lethal with *rad52Δ* but not with other DSB repair mutations such as *rad51Δ* [24]. Hyper-recombinant *rad3-102* cells, in which replication-born DSBs have been shown to accumulate, share a similar pattern of synthetic lethality with *rad52Δ* and MRX deletion but not with *rad51Δ* [25]. It is possible that this similarity is due to the formation of replication-born DSBs, which are repaired by Rad52/MRX-dependent HR that can be completed by two different pathways, one dependent on Rad51, the other on the Pol32 subunit of the replicative Pol∂ [25], which suggests a BIR-type of DSB repair [26]. Here we show that indeed *hst3Δ hst4Δ* double mutants are inviable or very sick in the absence of Rad51 if Pol32 is ablated (Figure 8). Therefore, H3K56 acetylation/deacetylation dynamics is critical to channel repair of replication-born DSBs into SCR as well as to prevent replication fork breakage that would make HR essential to reconstitute the fork by two alternative Rad52- and MRX-dependent pathways of repair with a differential dependency on Rad51 or Pol32.

## Discussion

We identified a number of proteins with a specific role in SCR that include Wss1, a SUMO or Ub-SUMO protease, and several proteins involved in chromatin remodeling, as Ahc1 (structural subunit of ADA histone acetyltransferase complex) and Hst3 or Rtt109, involved in acetylation/deacetylation of histone H3 lysine K56 (H3K56). These functions are necessary for the repair of replication-born DSBs by SCR. Mutations in the histone H3K56 residue to A, R and Q reveal that H3K56 acetylation/deacetylation is critical to promote SCR. This is the first evidence that chromatin marks can be used for the choice of repair template as a mechanism to warrant genome integrity, uncovering new functions for chromatin remodeling in genome dynamics. In addition, our study shows that Rad52 has specific residues with a key role in SCR but little or no impact on DSB repair via HR between homolog chromosomes, as deduced from the analysis of the *rad52-C180A* mutant.

The role for the SUMO or Ub-SUMO protease Wss1 in SCR [16] is particularly intriguing, given the known relevance of SUMOylation in different DSB repair pathways in yeast and mammals [27]; reviewed in [28]–[30], suggesting that SUMOylation may act at various steps and via different protein targets. Interestingly, a role for the Smc5-Smc6 complex containinig the Mms21/Nse2 SUMO ligase activity has been reported in SCR, even though the role of SUMOylation in this particular case has not been defined [31], [32]. It is also worth noting that Rad52 is indeed a target of SUMOylation that affects its DNA repair ability [33], [34].

Irc4, Irc9 and Irc19 are new proteins involved in SCR, as well as Bud27 and Pdr10, two proteins involved in stress response. The biochemical function of these proteins is yet unknown and further investigation of them is required to define their role in SCR. Our study also revealed that proteins involved in chromatin remodeling, such as the Ahc1 and Ada2 subunits of the ADA histone acetyl-transferase of the SAGA complex is important for SCR [17]. One of the functions of SAGA is transcriptional, in particular in transcription of RNA polII genes [35]. Perhaps these results imply a possible interconnection between transcription and DNA metabolism via a transcription-dependent chromatin remodeling, which is an interesting possibility.

A major focus of this work has been on the role of histone H3K56 acetylation/deacetylation in SCR. In *S. cerevisiae* acetylation of H3K56 (H3K56ac) occurs on newly synthesized histone H3 molecules by Rtt109 acetyl-transferase, facilitating their deposition onto newly replicated DNA during S phase, but disappears rapidly by the action of sirtuins Hst3 and Hst4 when cells enter G2/M [6], [7], [36]. Their deposition also increases in response to DNA damage in S phase [9], [11]. Strains lacking an acetylatable histone H3K56 show genetic instability and sensitivity to a subset of genotoxic agents including camptothecin (CPT) [6], [12]. This phenotype is possibly due to a key role of this modification in nucleosome assembly following DNA replication and DNA repair [8], [37]. Indeed, in agreement with our results implying a function in SCR, it has been recently suggested that H3K56 acetylation in nascent chromatin is important to complete the repair of DNA lesions and/or DNA replication [38]. As with other mutations affecting chromatin assembly, hyper-recombination can be explained by defective replication fork progression that would lead to DNA breaks (see [39]).

We show that *hst3* and *hst4* mutations specifically impair SCR. Given the redundancy of the two deacetylases, the synergistic effect of the mutations in the accumulation of Rad52 foci and the defect in SCR demonstrates that histone H3 deacetylation is critical in SCR and genome stability. Furthermore, the analysis of specific A, R and Q mutations of H3K56 that mimic either hyper-acetylation or deacetylation strengthens the notion that this mark is important for efficient SCR and for preventing genome instability. The relevance of histone H3K56 acetylation/deacetylation dynamics in genome instability has also been reported in mammalian cells for p300/CBP H3K56 acetyl-transferase and SIRT1 deacetylase [9], [11]. We propose that the histone H3K56 acetylation/deacetylation profile serves as a cell marker to favor SCR *versus* other mechanisms of repair of replication-born DSBs. It is worth noting that the effect of *asf1Δ*, which also impairs H3K56 acetylation [36], may be different as *asf1Δ* mutants are weakly affected in SCR at the early time points of the reaction [40], likely due to its function in other processes such as the DNA damage checkpoint [41].

**Figure 9:**
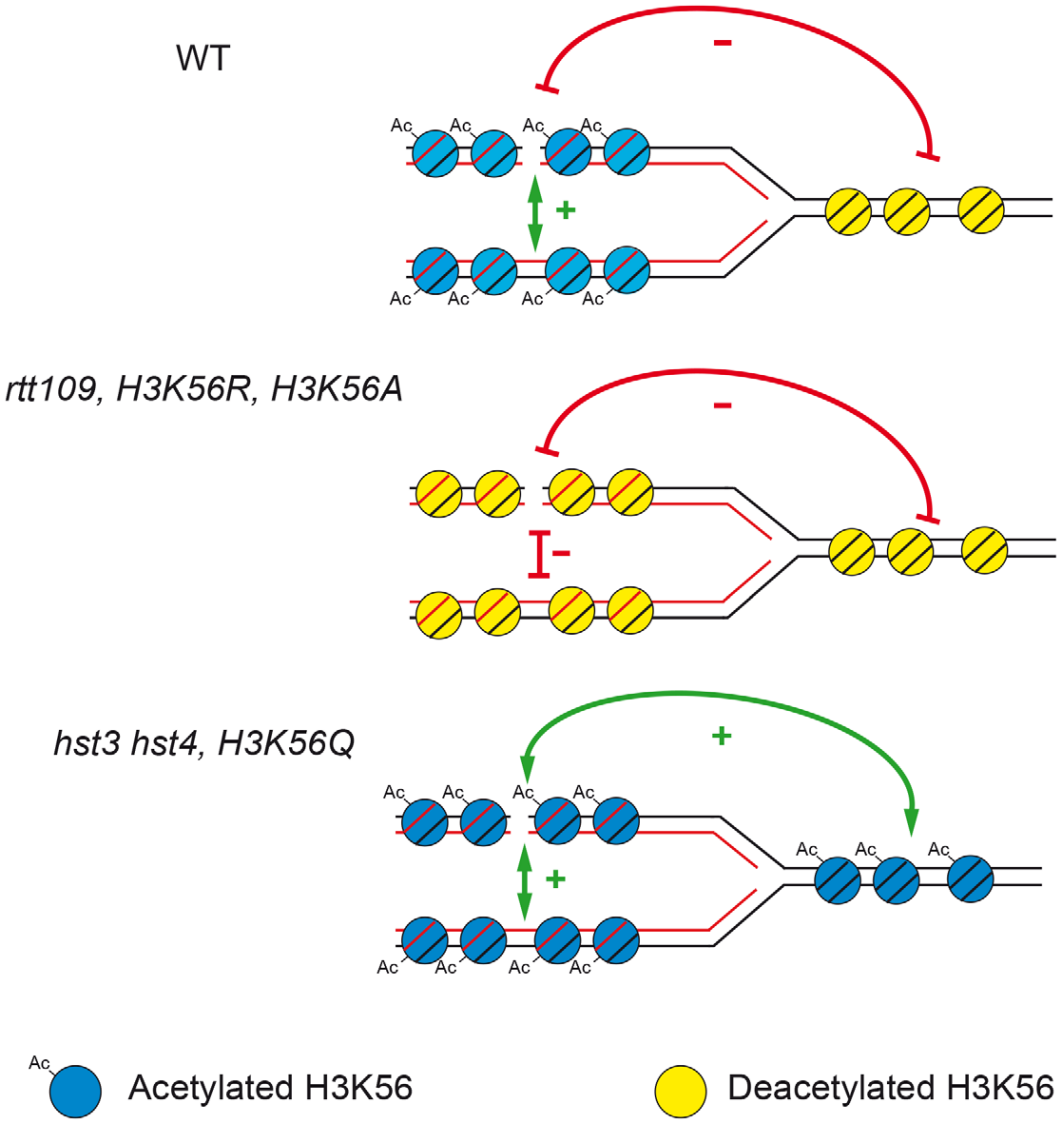
Model to explain how the state of acetylation/deacetylation of H3K56 influences SCR. Newly incorporated Histone H3 in the newly born sister-chromatids are acetylated, whereas the unreplicated DNA contains deacetylated histone H3. In the absence of K56 acetylation or when all histones H3 are acetylated, the recombination apparatus does not efficiently recognize the sister and the SCR preference is lost.

One of the known functions of histone H3K56 acetylation/deacetylation in chromatin dynamics during replication [6], [8], [24] is that acetylated histone H3 is incorporated into newly synthesized chromatin behind the replication fork, whereas deacetylated “old” histones are ahead of the fork. Here, we propose a model, depicted in Figure 9, to explain its role in favoring the choice for the sister as the preferential repair template for replication-generated DSBs. The preference for the sister for DSB repair is lost if H3K56 is deacetylated on both sides of the fork or hyper-acetylated. Deacetylated chromatin is involved in silencing and chromatin condensation [6], [42], [43], which may also explain the decreased efficiency of repair observed here due to limited accessibility of DNA repair proteins. It could also be that absence of H3K56 acetylation causes a defect in nucleosome assembly responsible for an impairment of SCR or negatively affects loading of cohesins, which has been shown to be required for SCR [4]. Nevertheless, the fact that H3K56 acetylation causes similar effects on SCR than H3K56 deacetylation or the *rad52-C180A* mutation (see below) makes rather unlikely that cohesin loading is the major cause of the SCR impairment. Therefore, the asymmetry of the acetylation state around the fork may facilitate the repair of a broken chromatid with its sister.

The intimate link of repair with chromatin modifications suggests that particular recombination proteins may have a differential capacity to interact with differently modified histones. In this sense, the existence of specific *rad52* alleles, known as class C mutants, which are defective in the repair of DSBs but proficient in spontaneous recombination (Figure 7) [15], [44], is particularly intriguing. In this work we demonstrate, using the *rad52-C180A* allele, that this phenotype is explained by a defect in SCR (Figure 2 and Figure 3). It would be interesting to see whether specific mutations in the early acting HR protein Rad52 might impair its ability to recognize different states of acetylated/deacetylated histone H3, therefore randomizing the template choice. Nevertheless, this is just one possibility as it is also plausible that a number of Rad52 residues, likely those identified in the class C alleles, play a role in favoring the sister as the main repair template choice by either facilitating interaction with some components of the sister such as particular histone residues, cohesins, etc. which would be worth investigating in the future.

Finally, our work provides genetic evidence for two HR pathways to reconstitute replication forks via SCR. We find that *hst3Δ hst4Δ* mutants are lethal with *rad52Δ* but not with *rad51Δ* unless the Pol32 subunit of Pol∂ is ablated ([24]; Figure 8). The same is observed in *rad3-102* mutants that accumulate single-strand DNA nicks that precede DSBs occurring by replication fork breakage [25]. These observations support a model of two mitotic Rad52/MRX-dependent mechanisms of SCR for the repair of replication-born DSBs, one being Rad51-dependent and the other Pol32-dependent [45], even though a synergistic effect caused by a masked role of Po32 in replication cannot be discarded.

In summary, our work provides new insights into SCR as a major mechanism of repair of replication-born DNA breaks. It shows the existence of factors and specific protein residues that play a role in the choice of the sister chromatid as the DNA repair template. These functions include the state of histone H3 K56 acetylation/deacetylation or specific DSB repair proteins acting at the early steps of homologous recombination such as Rad52. Importantly, our study demonstrates that failure to repair a replication-born DSB with the sister can lead to genome instability, raising new questions about the mechanisms by which DSB repair proteins and chromatin interact to favor one DSB repair pathway versus another.

## Materials and Methods

### Strains and plasmids

Yeast strains used in this work are listed in Table S1. All strains are in the W303 (WS strains) background with the only exception of *ada2Δ* (BY4741 background). Plasmids pRS316TINV and pCM189-L2HOr containing a 24-bp mini-HO site at the *Eco*RI internal site of *LEU2* were described previously [46]. Plasmid pWJ1344 was used for analysis of Rad52-GFP foci as described [47].

### Physical analysis of sister chromatid recombination

Sister chromatid recombination assays were carried out essentially as described [3]. Briefly, cells carrying pRS316-TINV were grown to mid-log phase in SC-Ura 3% glycerol 2% lactate; then, galactose (2%) was added to induce HO expression. Samples were collected at different time points and DNA was purified, digested with *Spe*I*-Xho*I, and analyzed by Southern using Hybond N+ (GE Healthcare) membranes. A ^32^P-labeled 0.22-kb *LEU2* probe was obtained by PCR using the primers 5′-GTTCCACTTCCAGATGAGGC-3′ and 5′-TTAGCAAATTGTGGCTTGA-3′. Quantification of DSBs (1.4-kb plus 2.4-kb bands) and SCR (4.7-kb band) relative to the total DNA was calculated with a Fuji FLA-5100. Each experiment was done in triplicate, but one representative is shown.

### Genetic and molecular analysis of recombination

The analysis of HO-mediated DSB recombination both with TINV and plasmid-chromosome system *leu2HOr/leu2-k* was performed as described previously [46], [48]. Briefly, mid-log phase yeast cells carrying the HO gene under the control of *GAL1* were obtained from SC-3% glycerol-2% lactate liquid cultures and split into two halves. One-half was maintained in liquid SC-3% glycerol/2% lactate + dox (no HO expression) and the other was cultured in SC-2% galactose + dox for 5 hr (HO expression). Recombinants were selected on SC-leu-ura containing 2% glucose. The chromosomal direct-repeat system *his3-*Δ*5′::his3-*Δ*3′* [23] was used to analyze unequal sister chromatid recombination. In this system, recombinants were selected on SC-His containing 2% glucose. In all cases, recombination frequencies are the median values of fluctuation tests performed with six independent yeast colonies each, as previously described [46]. For every genotype, fluctuation tests were repeated three times with three different yeast transformants. The final frequency shown for each genotype corresponds to the mean value of the three median frequencies obtained from the tests.

### Miscellanea

For the analysis of HU and MMS sensitivity, cells were grown to mid-logarithmic phase in YPD to a final concentration of 0.5×10^7^ cells/ml and 10-fold serial dilutions were spotted onto YPD plates containing different concentrations of MMS or HU at 30°C. Rad52 foci were determined in S/G2 cells as described [49].

## Supporting Information

Figure S1 Representative Southerns of different kinetic experiments of DSB repair via SCR in isogenic wild-type (W303) and hiperecombinogenic mutants at 0 h, 3 h, 6 h and 9 h after HO induction. Asterisks mark bands corresponding to endogenous *LEU2* gene.
(TIF)

Figure S2 Quantification of the percentage of DSBs generated in pRS316-TINV at different times after HO induction in 2% galactose from the experiments of Figure S1.
(TIF)

Figure S3 Quantification of the percentage of SCR intermediates generated in pRS316-TINV at different times after HO induction in 2% galactose from the experiments of Figure S1.
(TIF)

Figure S4 Physical analysis of SCR in isogenic wild-type and *ada2*Δ strains. Others details as in Figure 1 and Figure 3.
(TIF)

Figure S5 Effect of the changes in the state of H3K56 acetylation in resistence to genotoxic agents. Effect of H3K56 acetylation/deacetylation mutants in the sensitivity to HU and MMS of isogenic wild-type (WS), *hst3*Δ, *hst4*Δ, *hst3*Δ *hst4*Δ and *H3K56R*, *H3K56A*, *H3K56Q* strains. Growth of 10-fold serial dilutions of mid-log phase cultures of the WT and isogenic mutant strains (WS) and a *rad52*Δ control is shown on YPD plates containing HU or MMS is shown.
(TIF)

Table S1 Strains used in this study.
(DOC)
