# Supplementary figures and images for: Histone H3K56 Acetylation, Rad52, and Non-DNA Repair Factors Control Double-Strand Break Repair Choice with the Sister Chromatid
Source: PLoS Genet. 2013 Jan 24;9(1):e1003237. doi: 10.1371/journal.pgen.1003237 (PMC3554610; doi:10.1371/journal.pgen.1003237)

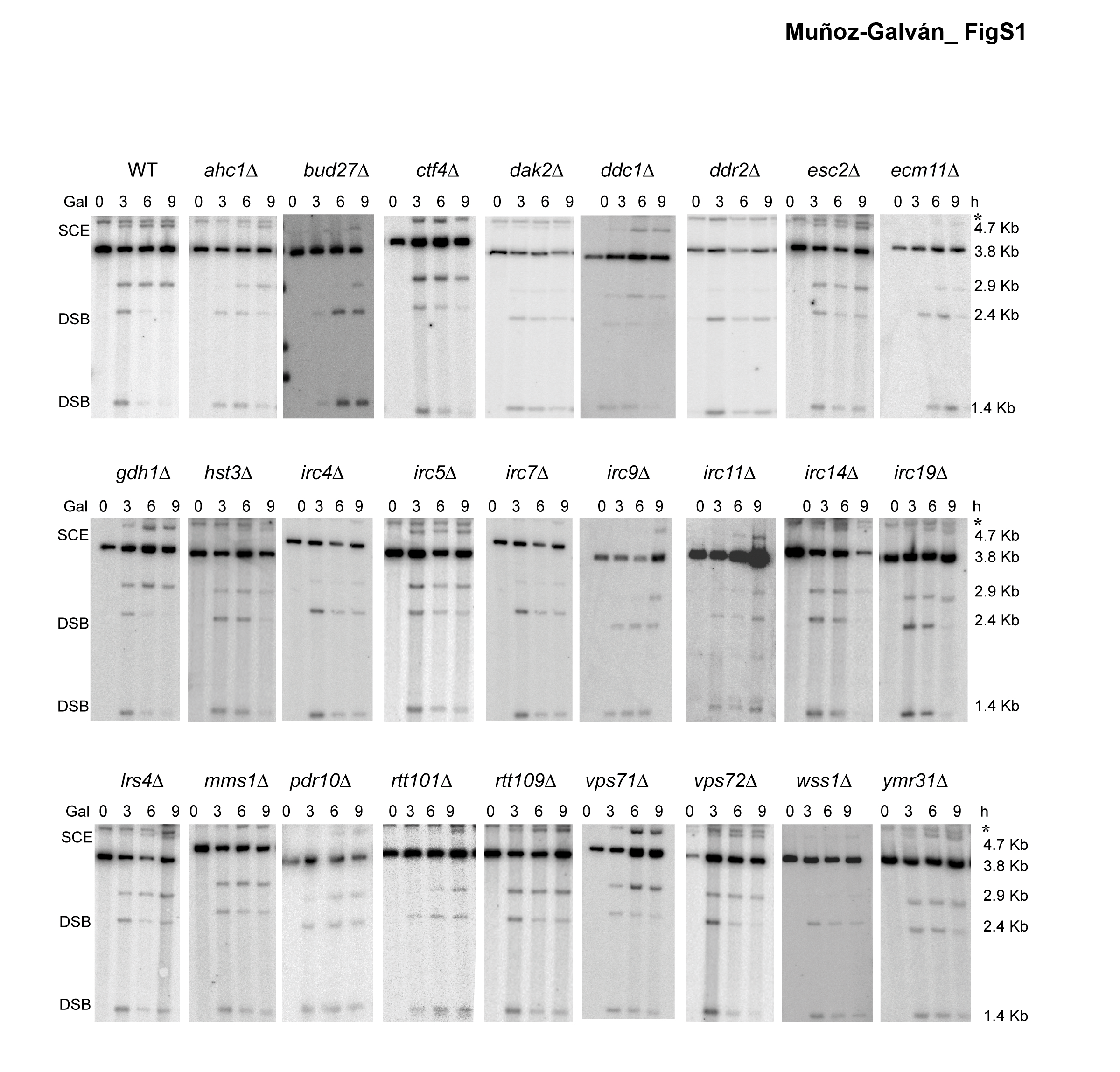

Supplement: Figure S1 — Representative Southerns of different kinetic experiments of DSB repair via SCR in isogenic wild-type (W303) and hiperecombinogenic mutants at 0 h, 3 h, 6 h and 9 h after HO induction. Asterisks mark bands corresponding to endogenous LEU2 gene. (TIF) [file pgen.1003237.s001.tif]

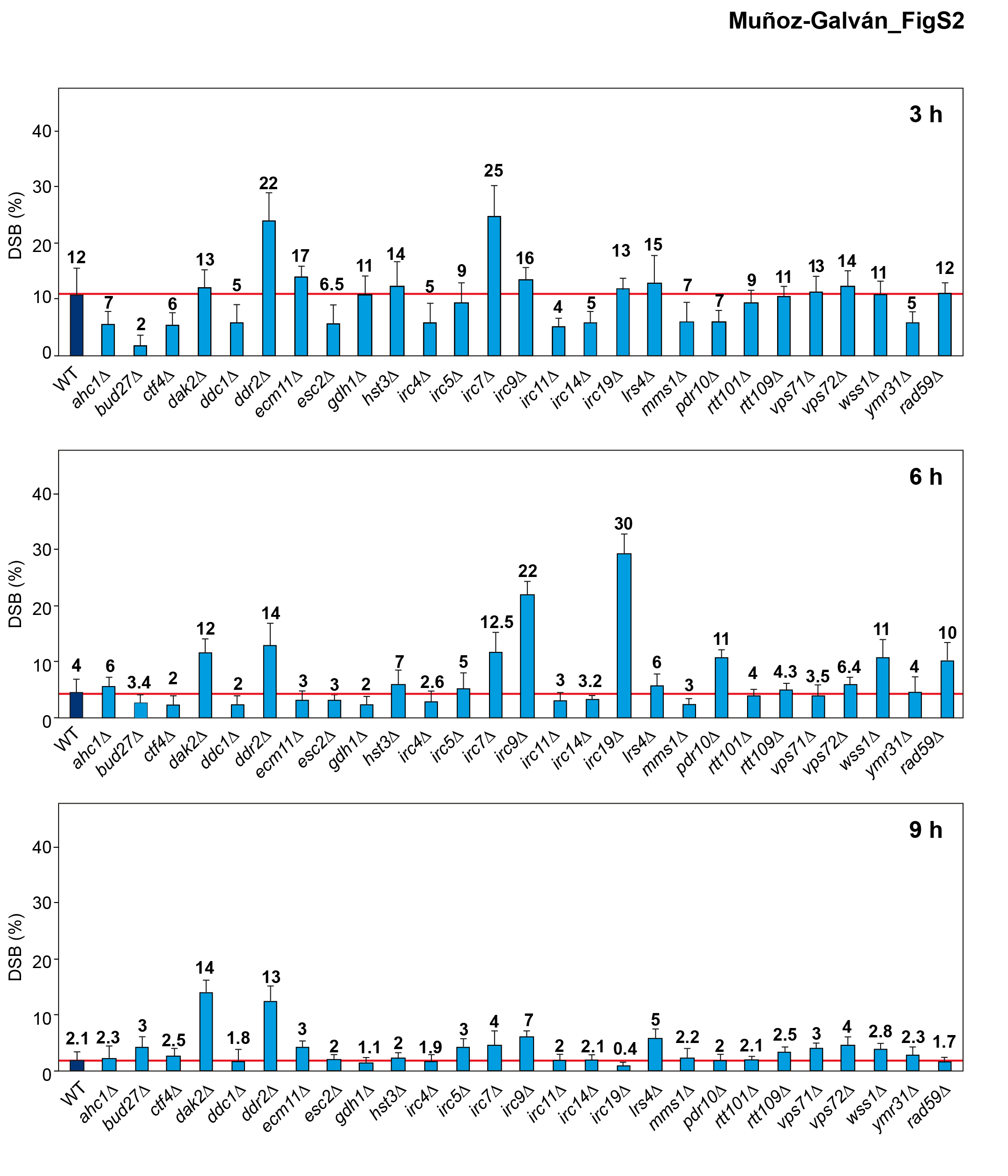

Supplement: Figure S2 — Quantification of the percentage of DSBs generated in pRS316-TINV at different times after HO induction in 2% galactose from the experiments of Figure S1. (TIF) [file pgen.1003237.s002.tif]

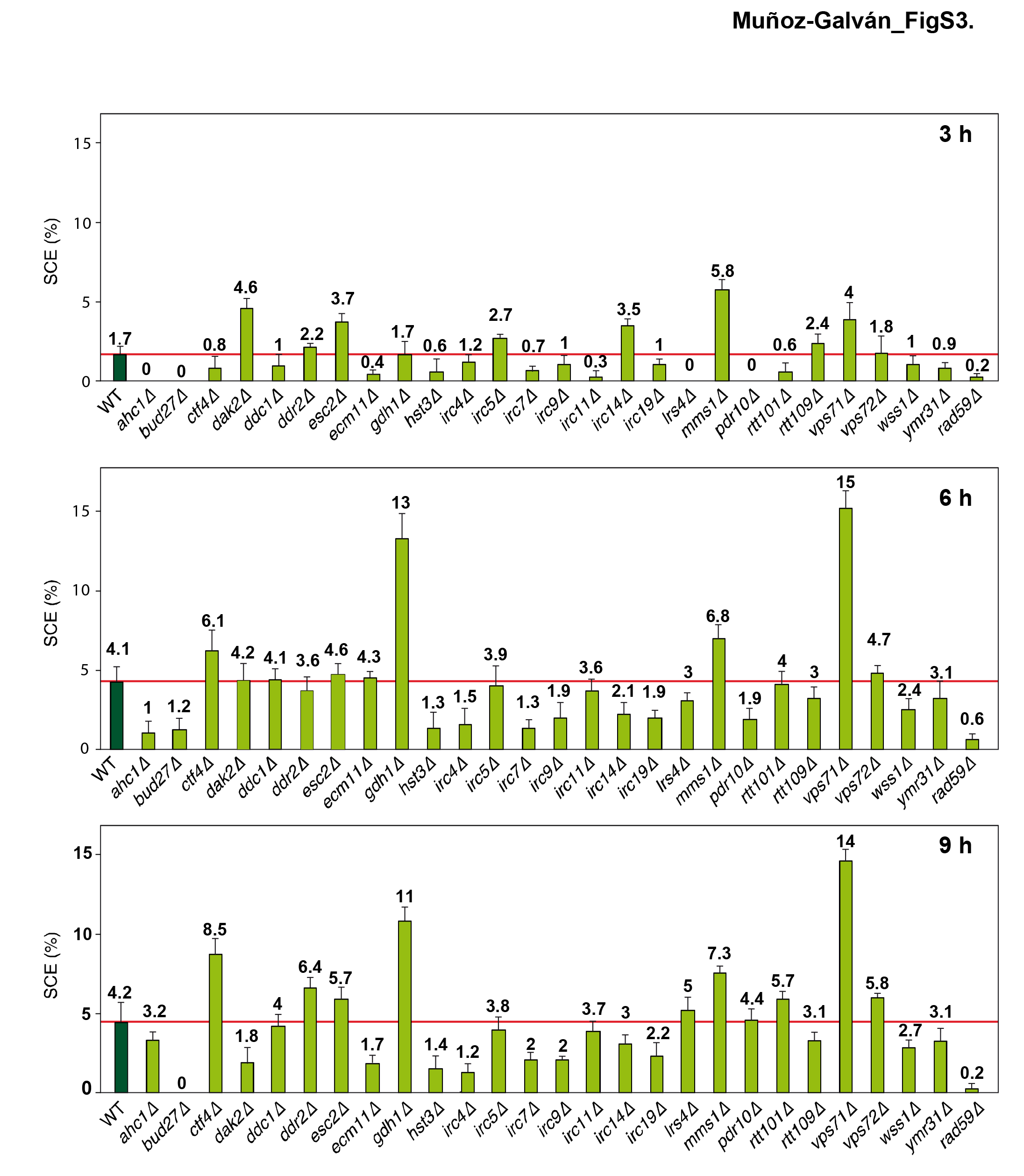

Supplement: Figure S3 — Quantification of the percentage of SCR intermediates generated in pRS316-TINV at different times after HO induction in 2% galactose from the experiments of Figure S1. (TIF) [file pgen.1003237.s003.tif]

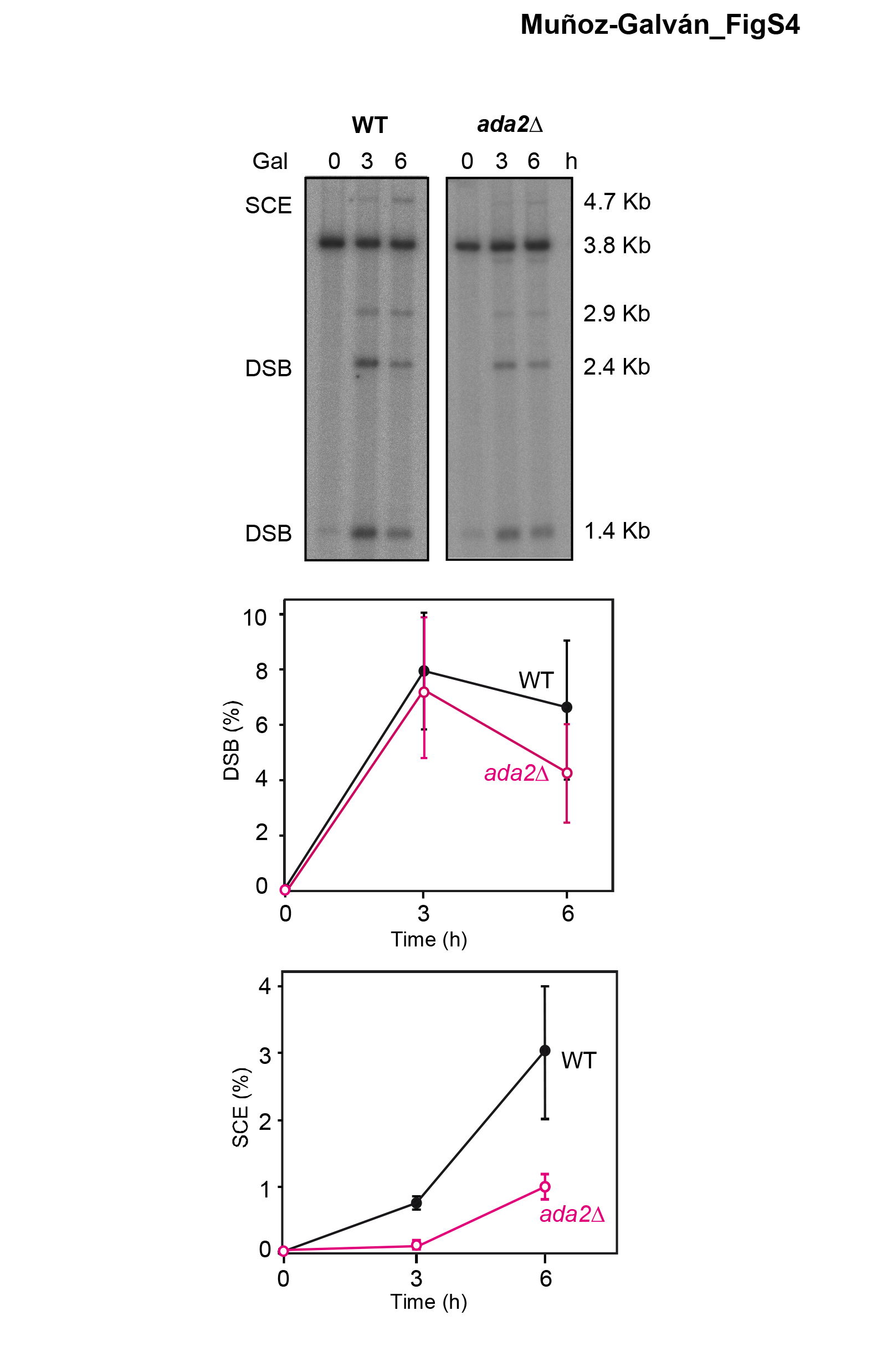

Supplement: Figure S4 — Physical analysis of SCR in isogenic wild-type and ada2Δ strains. Others details as in Figure 1 and Figure 3. (TIF) [file pgen.1003237.s004.tif]

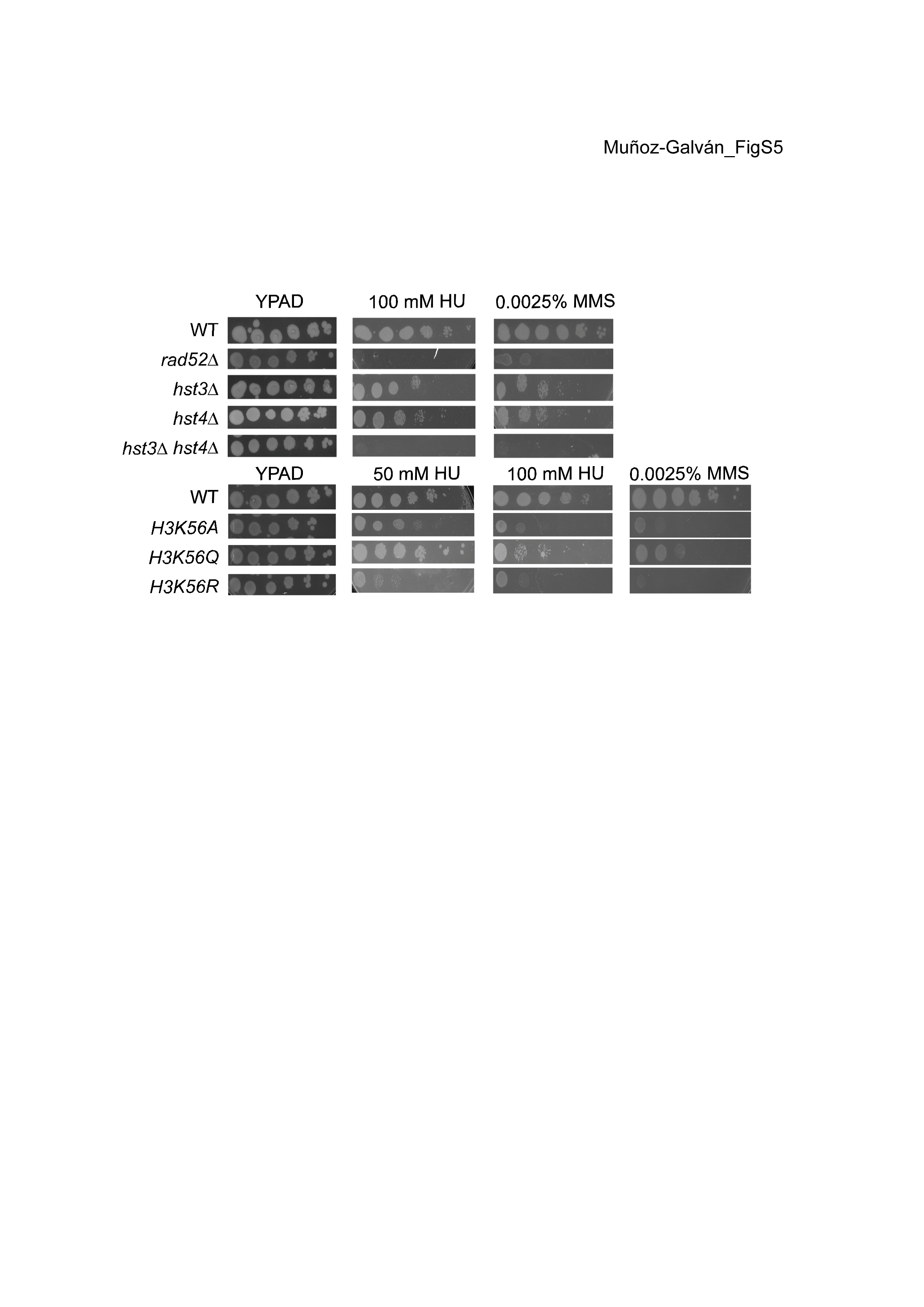

Supplement: Figure S5 — Effect of the changes in the state of H3K56 acetylation in resistence to genotoxic agents. Effect of H3K56 acetylation/deacetylation mutants in the sensitivity to HU and MMS of isogenic wild-type (WS), hst3Δ, hst4Δ, hst3Δ hst4Δ and H3K56R, H3K56A, H3K56Q strains. Growth of 10-fold serial dilutions of mid-log phase cultures of the WT and isogenic mutant strains (WS) and a rad52Δ control is shown on YPD plates containing HU or MMS is shown. (TIF) [file pgen.1003237.s005.tif]
