# Supplementary material for: Histone H3K56 Acetylation, Rad52, and Non-DNA Repair Factors Control Double-Strand Break Repair Choice with the Sister Chromatid
Source: PLoS Genet. 2013 Jan 24;9(1):e1003237. doi: 10.1371/journal.pgen.1003237 (PMC3554610; doi:10.1371/journal.pgen.1003237)
Supplement: Table S1 — Strains used in this study. (DOC) [file pgen.1003237.s006.doc]

**Table SI.** List of strains used in this study.

| Strain | Genotype | Source |
| --- | --- | --- |
| W303-1A | *MAT***a** *leu2-3,112 trp1-1 ura3-1 ade2-1 his3-11,15 can1-100* | This study |
| W6345-1A | *MAT***a** *ura3-1* *trp1-1 his3-11,15 can1-100 ADE2* *LYS2* *ahc1∆::KanMX4* *leu2*∆EcoRI | [12] |
| W6331-1B | *MAT***a** *ura3-1* *trp1-1 his3-11,15 can1-100 ADE2* *LYS2* *esc2∆::KanMX4* *leu2*∆EcoRI | [12] |
| W6332-11B | *MAT***a** *ura3-1* *trp1-1 his3-11,15 can1-100 ADE2* *LYS2* *hst3∆::KanMX4* *leu2*∆BstEII | [12] |
| W6364-11B | *MAT*α *ura3-1* *trp1-1 his3-11,15 can1-100 ADE2* *LYS2* *irc5∆::KanMX4* *leu2*∆BstEII | [12] |
| W6353-20A | *MAT***a** *ura3-1* *trp1-1 his3-11,15 can1-100 ADE2* *LYS2* *lrs4∆::KanMX4* *leu2*∆EcoRI | [12] |
| W6406-9A | *MAT*α *ura3-1* *trp1-1 his3-11,15 can1-100 ADE2* *LYS2* *mms1∆::KanMX4* *leu2*∆BstEII | [12] |
| W7495-11B | *MAT***a** *leu2-3,112 ura3-1* *trp1-1 his3-11,15 can1-100 ADE2* *LYS2* *ctf4∆::KanMX4* | [12] |
| W4818-1C | *MAT*α *leu2-3,112 ura3-1* *trp1-1 his3-11,15 can1-100 ADE2* *LYS2* *rtt109∆::KanMX4* *RAD52-YFP* | [12] |
| W6676-2D | *MAT***a** *ura3-1* *trp1-1 his3-11,15 can1-100 ADE2* *LYS2* *vps72∆::KanMX4* *leu2*∆EcoRI | [12] |
| W6413-1D | *MAT***a** *ura3-1* *trp1-1 his3-11,15 can1-100 ADE2* *LYS2* *bud27∆::KanMX4* *leu2*∆EcoRI | [12] |
| W6322-17D | *MAT*α *ura3-1* *trp1-1 his3-11,15 can1-100 ADE2* *LYS2* *dak2∆::KanMX4* *leu2*∆BstEII | [12] |
| W6348-21B | *MAT*α *ura3-1* *trp1-1 his3-11,15 can1-100 ADE2* *LYS2* *ddr2∆::KanMX4* *leu2*∆BstEII | [12] |
| W6327-2D | *MAT***a** *ura3-1* *trp1-1 his3-11,15 can1-100 ADE2* *LYS2* *ecm11∆::KanMX4* *leu2*∆EcoRI | [12] |
| W6315-5C | *MAT***a** *ura3-1* *trp1-1 his3-11,15 can1-100 ADE2* *LYS2* *irc9∆::KanMX4* *leu2*∆EcoRI | [12] |
| W6321-8A | *MAT***a** *ura3-1* *trp1-1 his3-11,15 can1-100 ADE2* *LYS2* *irc11::KanMX4* *leu2*∆EcoRI | [12] |
| W6424-6B | *MAT***a** *ura3-1* *trp1-1 his3-11,15 can1-100 ADE2* *LYS2* *irc14::KanMX4* *leu2*∆BstEII | [12] |
| W7257-16D | *MAT***a** *ura3-1* *trp1-1 his3-11,15 can1-100 ADE2* *LYS2* *irc19∆::KanMX4* *leu2*∆BstEII | [12] |
| W6351-17B | *MAT***a** *ura3-1* *his3-11,15 lys2D can1-100 rad5-G535R* *TRP1 ADE2* *pdr10∆::KanMX4* *leu2*∆EcoRI | [12] |
| W7674-2C | *MAT***a** *leu2-3,112 ura3-1* *his3-11,15 can1-100 TRP1 ADE2* *LYS2* *wss1∆::KanMX4 RAD52-YFP* | [12] |
| W6398-9B | *MAT*α *ura3-1* *trp1-1 his3-11,15 can1-100 ADE2* *LYS2* *ymr31∆::KanMX4* *leu2*∆BstEII | [12] |
| W6324-8A | *MAT***a** *ura3-1* *trp1-1 his3-11,15 can1-100 ADE2* *LYS2* *ddc∆:::KanMX4* *leu2*∆BstEII | [12] |
| W6244-4C | *MAT***a** *leu2-3,112 trp1-1 ura3-1 ade2-1 his3-11,15 can1-*  *100 vps71∆::LEU2* | [12] |
| W6243-11A | *MAT***a** *ura3-1* *trp1-1 his3-11,15 can1-100 ADE2* *LYS2* *gdh1∆::KanMX4* *leu2*∆EcoRI | [12] |
| W6311-3D | *MAT***a** *ura3-1* *trp1-1 his3-11,15 can1-100 ADE2* *LYS2* *irc4∆::KanMX4* *leu2*∆EcoRI | [12] |
| U1952 | *MAT*a *leu2-3,112 ura3- MAT*α *leu2-3,112 ura3-1* *his3-11,15 can1-100 lys2∆* *ADE2* *TRP1 rtt101DkanM* | [12] |
| W6357-12C | *MAT***a** *ura3-1* *trp1-1 his3-11,15 can1-100 ADE2* *LYS2* *irc7∆::KanMX4* *leu2*∆EcoRI | [12] |
| WLK | *MAT***a**-*inc* *trp1-1 leu2 k ura3 ade2 his3∆ ade3::gal-HO* | This study |
| WLK-L4 | WTLK *lrs4∆::KanMX4* | This study |
| WLK-W1 | WTLK *wss1∆::KanMX4* | This study |
| WLK-B27 | WTLK *bud27∆::KanMX4* | This study |
| WLK-P10 | WTLK *pdr10∆:: KanMX4* | This study |
| WLK-I9 | WTLK *irc9∆:: KanMX4* | This study |
| WLK-I14 | WTLK *irc14∆::KanMX4* | This study |
| WLK-I19 | WTLK *irc19∆::KanMX4* | This study |
| WLK-I4 | WTLK *irc4∆::KanMX4* | This study |
| WLK-I7 | WTLK *irc7∆::KanMX4* | This study |
| WLK-H3 | WTLK *hst3∆::KanMX4* | This study |
| WLK-RT9 | WTLK *rtt109∆::KanMX4* | This study |
| WLK-AH1 | WTLK *ahc1∆::KanMX4* | This study |
| WLK-H4 | WTLK *hst4∆::KanMX4* | This study |
| WLK-H34 | WTLK *hst4∆::KanMX4 hst3∆::KanMX4* | This study |
| WLK-C180A | WTLK *rad52-C180A* | This study |
| WS | *MAT***a**-*inc* *trp1-1 ura3-1 ade2-1 his3-11,15 can1-100 ade3::gal-HO leu2::SFA1* | This study |
| WS-52 | WS *rad52∆::KanMX4* | This study |
| WS-C180A | WS *rad52-C180A* | This study |
| WS-I19 | WS *irc19∆::KanMX4* | This study |
| WS-I14 | WS *irc14∆::KanMX4* | This study |
| WS-I4 | WS *irc4∆::KanMX4* | This study |
| WS-L4 | WS *lrs4∆:::KanMX4* | This study |
| WS-E11 | WS *ecm11∆::KanMX4* | This study |
| WS-H3 | WS *hst3∆:: KanMX4* | This study |
| WS-H4 | WS *hst4∆:: KanMX4* | This study |
| WS-H34 | WS *hst3∆::KanMX4 hst4∆::KanMX4* | This study |
| WS-RT9 | WS *rtt109∆::KanMX4* | This study |
| WS-AH1 | WS *ahc1∆::KanMX4* | This study |
| WS-W1 | WS *wss1∆::KanMX4* | This study |
| WS-B27 | WS *bud27∆::KanMX4* | This study |
| WS-P10 | WS *pdr10∆::KanMX4* | This study |
| WS-I7 | WS *irc7∆::KanMX4* | This study |
| WS-I9 | WS *irc9∆::KanMX4* | This study |
| HMY133 | *MAT***a** *trp1-1 ura3-1 his3-11,15 leu2-3,112 ade2-1 can1-100 hht1-hhf1::LEU2 hht2-hhf2::KanMX3* [*YCp22 HHT1 HHF1 TRP1*] | [6] |
| HMY134 | *MAT***a** *trp1-1 ura3-1 his3-11,15 leu2-3,112 ade2-1 can1-100 hht1-hhf1::LEU2 hht2-hhf2::KanMX3* [*YCp22 hht1* K56A *HHF1 TRP1*] | [6] |
| HMY135 | *MAT***a** *trp1-1 ura3-1 his3-11, 15 leu2-3,112 ade2-1 can1-100 hht1-hhf1::LEU2 hht2-hhf2::KanMX3* [*YCp22 hht1* K56Q *HHF1 TRP1*] | [6] |
| HMY136 | *MAT***a** *trp1-1 ura3-1 his3-11,15 leu2-3,112 ade2-1 can1-100 hht1-hhf1::LEU2 hht2-hhf2::KanMX3* [*YCp22 hht1* K56R *HHF1 TRP1*] | [6] |
| YNN299 | *MAT***a** *his3Δ200 ura3-1 lys2-801 ade2-101 his3-Δ3 ́his3-Δ5 ́:: URA3* | [23] |
| YNN-H3 | *YNN299 hst3∆::KanMX4* | This study |
| YNN-AH1 | *YNN299* *ahc1∆::KanMX4* | This study |
| YNN-RT9 | *YNN299* *rtt109∆::KanMX4* | This study |
